# Why do I need to belong? Black women and Latinas navigate medical education beyond belonging toward rightful presence

**DOI:** 10.1186/s12909-025-07781-9

**Published:** 2025-08-27

**Authors:** Sacha Sharp, Ashley Hixson Clarke, Oseme Precious Okoruwa

**Affiliations:** 1 Vice Chair for Strategic Engagement, Department of Medicine, Indiana University School of Medicine, 545 Barnhill Dr., EH 305, Indianapolis, IN 46202 USA; 2 Equity, Inclusion, and Belonging, The Center on Budget and Policy Priorities, 1275 First Street NE, Suite 1200, Washington, DC USA; 3 Indiana University School of Medicine, Indianapolis, IN, Indiana 46202 USA

**Keywords:** Black women, Latinas, Medical education

## Abstract

**Background:**

Medical education research indicates that students of color face discrimination affecting their sense of belonging within historically white institutions (HWIs) of academic medicine. This is especially true for the students with intersectional identities such as Black women and Latinas, who continue to make up a small percentage of medical school matriculants each year.

**Methods:**

In 2021, we initiated a virtual community of seven Black women and Latina medical students from around the midwestern United States. Over a two-year period, participants engaged in online message boards and research interviews discussing their experiences for the purpose of promoting a sense of belonging. Using the data collected from the virtual community, we implemented a composite narrative methodology undergirded by theoretical frameworks Black Feminist Thought (BFT) and Latina Chicana Feminism (LCFT) to answer our research question. The purpose of the study was to understand the impact of a sense of belonging on participant experiences in medical school, while also protecting their anonymity.

**Results:**

The findings for this study are illustrated via composite narratives that are organized as a conversation between a Black woman and a Latina medical student named Moon and Star. The composite narratives highlight experiences had among the seven participants demonstrating how minoritized identity development is inconsistent with belonging and the need for HWIs of academic medicine to establish a rightful presence beyond belonging. Rightful presence is a concept where the qualities and characteristics of an individual are accepted in an environment and therefore, do not require the individual to adjust for purposes of belonging.

**Conclusion:**

This study identified rightful presence as a way to engage Black women and Latina students in medical education. Based on the findings from this study we encourage moving beyond a sense of belonging approach toward welcoming the unique contributions of Black women and Latinas starting with their right to be in medical education spaces.

**Supplementary Information:**

The online version contains supplementary material available at 10.1186/s12909-025-07781-9.

## Background

Students identified as underrepresented in medicine (URiM) are often overwhelmed and discouraged by their negative experiences at historically white institutions (HWIs) of academic medicine [1–3]. For URiM students to attend HWIs of academic medicine often means overcoming the disadvantages associated with entering spaces not designed to foster their cultural capital [4]. Consistently, URiM students are forced to navigate harassment and discrimination in addition to maintaining academic success [5–7]. In response, medical educators have embraced concepts such as a sense of belonging to engage URiM students and support them in achieving academic success [8]. According to Hurtado & Carter, a sense of belonging is defined as being connected with the institution and having a shared sense of purpose with that organization [9]. In general, a sense of belonging indicates that a student fits with a particular education environment and fits in with their peers through a psychological sense of identification and affiliation [10].

Unfortunately, URiM students continue to experience challenges even when medical educators put forth efforts toward creating a sense of belonging. Specifically, Black women and Latina medical students experience alienation and isolation, challenges that often go unheard because of the intersectional nature of their identity [11]. Similarly, Black women and Latinas make up a lower percentage of medical student matriculants at 4.5% and 3.3%, respectively [12]. The confluence of their ethnic, racial, and gendered experiences goes unacknowledged because they are often grouped with all members of the URiM student population. The purpose of this research was to unearth those intersectional experiences by exploring how Black women and Latinas navigated medical school to better understand how a sense of belonging may or may not have impacted that experience. Our research question was, how important is a sense of belonging to the medical school experiences of Black women and Latinas?

## Literature review

Significant research calls for diversifying medical education and creating equitable and inclusive environments [13–16]. It is understood that diversity contributes to health equity and that physicians from minoritized backgrounds contribute to instruction with culturally engaging pedagogies that support student success [17, 18]. However, medical schools are failing to increase diversity even when diversity is established as a pillar of educational excellence [16].

HWIs of academic medicine often maintain a whiteness culture that benefits men, prioritizing these identities as currency privileged by the institution [19]. These privileges are accommodated by what are referred to as “race-neutral” and “sex-neutral” policies and practices that relegate non-white and non-male identities to lower positions with little representation [19].

Therefore, it is unsurprising that there is a dearth of research that explores the experiences of diverse medical students, specifically Black women and Latinas.

Historically, Black women have been subjected to stereotypes and archetypes that have harmful effects on how they are perceived and their social location in society [20]. As Black women enter and persist through the academy, they often experience gendered racism particularly in white and male-dominated spaces such as HWIs [21, 22]. Black women who choose to navigate these environments may face discrimination, isolation, and microaggressions that add to their intellectual and emotional labor and ultimately influence how they perform academically and socially [23, 24]. Although mired with nuance, stereotypical perceptions of gender roles and culture also play a critical part for Latinas in the academy [25].

Gendered roles prescribed to Latinas may require a balance between professional education and “fulfilling their roles within the family” [26]. Roles within the family include managing and maintaining cultural traditions, sibling or elder caregiving, and providing financial support [27].

According to Yosso*, cultural considerations for Latinas’ persistence in higher education include varying forms of capital [4]. These may include a sense of community based on family relationships and those on their college campus, and linguistic capital [4, 29, 30]. Although these forms of capital are valuable, they are traditionally viewed as deficits, often lead to marginalization [4], and are perceived negatively by HWIs [28].

When considering how Black women and Latinas experience medical education, it is important to understand how their intersectional identities contribute to their work and how they are perceived by their peers. For Black women in medicine, a culture of microaggressions and macroaggressions requires that Black women prove themselves and their abilities to their colleagues while bearing the psychological impacts of racism [31]. Black women also experience barriers to leadership, professional development, and equal compensation [31].

These challenges are not unique as Latina medical professionals also face barriers to career advancement due to discrimination in their training and in their workplaces [32]. Latinas in medical training experience considerably high rates of challenges around mental health with one study showing that rates of depression, burnout, and imposter syndrome were 76.2%, 87.4%, and 90.7%, respectively, among those surveyed in this population [32]. These experiences are in direct contrast to the intentions of a sense of belonging. Thus, it is important to better understand how Black women and Latinas experience medical education with the intention of improving their experiences and being more accommodating of the capital these populations bring to the medical field.

### Reflexivity

Prior to discussing the theoretical framework for this study, we find it important to share our reflexivity to better contextualize how we positioned ourselves in the research. We acknowledge the importance of our identities as three Black women researchers who hold shared social identities with the participants (e.g., race, gender, class, economic status), which will be further discussed in the following section. However, we understand that we also may hold bias because of our various beliefs and experiences with HWIs of academic medicine. Through our research reflexivity, we reflected upon the uniqueness of the participant experiences while addressing our own biases and assumptions with what and how the participants’ narratives should be shared to maintain rigorous research standards. To do this, we engaged in self-reflection after the focus groups and a collaborative dialogue throughout the research process to highlight how assumptions about the data might influence our interpretation of the data.

### Theoretical framework

We used both Black Feminist Thought (BFT) and a concept derived from BFT called Latina/Chicana Feminist Perspective, or LCFP, as our theoretical frameworks [33, 34]. BFT is a theory used for understanding Black women’s experiences in the U.S. The theory is based on emphasizing the intersectional identities Black women hold that are rooted in race, gender, and class with relation to the structural and systemic oppression [33]. The theory promotes tenets that highlight Black women as intellectuals who can better navigate society when given the ability to commune with other Black women [35]. As members of the research team, we assume positionalities that are consistent with the use of this framework since the framework asserts the importance of researcher identity alignment for the purpose of accurately analyzing data.

Consistently, LCFP considers the intersectional identities of Latinas and Chicanas, and aims to center these women in their own knowledge creation. LCFP emphasizes how Latinas and Chicanas come to understand knowledge using concepts like Nepantla, El Mundo Zurdo, and Coyolxauhqu, which mean highlighting the experience of living between worlds, challenging oppressive norms, and emphasizing healing through language, respectively [36].

Both BFT and LCFP, center Black women and Latinas’ lived experiences and honor their perspectives by using their voices to define those experiences, and to create their own self-definitions [33]. By using BFT and LCFP we understand that Black women and Latinas are in the best position to clarify their standpoints when determining their needs for academic success in medical school. We also consider the idea of outsiders within [35], noting that Black women and Latinas often exist as outsiders within medical education environments where their experiences are made invisible. Taken together, BFT and LCFP empower Black women and Latinas to leverage their cultural intuition in historically white spaces, regardless of how they may be treated structurally and systemically. These frameworks recognize the professional and personal acuity of Black women and Latinas, preparing them to navigate the dominant culture.

Using these frameworks, we investigate how Black women and Latinas navigate medical education and interrogate the importance of a sense of belonging.

## Methodology

For this research we use composite narratives as the methodological approach. Composite narrative, or composite counter-storying, is a methodology developed in the field of sociology designed to describe an emotional situation without breaching the confidentiality or anonymity of the various individuals sharing their experiences [37]. Consistent with qualitative research, composite narratives allow researchers to use their judgement and expertise to analyze multiple datasets and present key findings. Consistently, composite narratives allow researchers to highlight findings through fictionalized narratives that combine several datasets into one retelling. In other words, composite narratives account for the complexity of the qualitative datasets and honor the multiple voices of participants by offering the essence of what was discussed in one story [37]. Lastly, counter narratives make information more accessible to audiences that may be unfamiliar with traditional concepts often presented in academic writing.

We are using composite narratives in this study to protect the identities of the medical students, now interns, who went into competitive fields of medicine. We agreed that the medical students shared controversial statements about their institutions and information about their personal experiences they considered private, such as their ability status. Using composite narratives, we conveyed the richness of the data without compromising the students’ status within their chosen field.

## Methods

In 2021, authors one and two established a virtual community (VC) for Black women and Latinas interested in Internal Medicine at academic medicine HWIs in the midwestern United States. VCs are online spaces where individuals who share interests and goals come together [38]. VCs incorporate message boards, information sharing, and videoconferencing for individuals to engage and communicate with a common purpose. In the medical field, online healthcare VCs are adapted to serve patients who are experiencing similar diseases and who are undergoing the same treatment [39, 40]. Informed by the Culturally Engaging Campus Environments (CECE) model [41], BFT, and LCFP, the digital environment was designed to be a culturally engaging virtual space for Black women and Latinas that promoted a sense of belonging, cross-cultural interactions, cultural validity, and holistic support, among other indicators found in CECE. The VC met monthly via Zoom over two years for community discussions and engagement. In addition, the women were encouraged to interact with one another via the digital environment, Slack, to chat informally or receive advice from members of the research team.

In addition to informal channels, which are outside the scope of this research study, we held four semi-structured focus group sessions. During the focus groups, author one asked questions about medical school experiences starting with pre-matriculation, while author two took notes. The focus groups lasted 60–90 min and occurred twice a year over the two-year period. At least four women were present at each focus group, and the sessions were recorded via Zoom. We also conducted one individual interview with each participant at the end of the VC to better understand their personal experiences with medical education. These individual interviews were also designed to close out the VC experience. The interviews lasted 60 min and they were also recorded via Zoom. All data was transcribed using Verbit.AI, a transcription and captioning service approved as a university vendor at author one’s home institution.

The participants were incentivized with $250 for their time over the two-year period, receiving $125 increments at the end of each year. They also received educational resources and other items they felt they needed to be successful in medical school, such as access to Internal Medicine question banks through UWorld. This project was expedited by the university Institutional Review Board and funded through an internal institutional grant.

### Participants

We recruited seven women to participate in the research study, a number consistent with phenomenology research [42]. The women were recruited nationally using purposeful snowball sampling via social media and URiM community listservs such as the Student National Medical Association. The participants were Black or Latina racially and identified ethnically as African American, Puerto Rican, Mexican American, or Bolivian. The participants were between the ages of 24 and 33 at the time of the study **(**Table 1**)**, and anticipated graduation from medical school in 2022, 2023, or 2024. The women also shared other identities (e.g., ability status) salient to their overall experience during the focus groups, information that will be shared in the findings section of this paper.

**Table 1:** Participant demographics

	Age	Race	Ethnicity	Sex	Sexual Orientation	Highest Education Level
Participant 1	24	Multiracial	Black	F	Heterosexual	Bachelor’s
Participant 2	26	Black or African American	Black	F	Lesbian	Master’s
Participant 3	26	Black or African American	African American	F	Heterosexual	Bachelor’s
Participant 4	27	Hispanic or Latino	Puerto Rican	F	Heterosexual	Bachelor’s
Participant 5	28	Hispanic or Latino	Bolivian	F	Bisexual	Master’s
Participant 6	28	Hispanic or Latino	Mexican	F	Heterofluid	Bachelor’s
Participant 7	33	Hispanic or Latino	Mexican American	F	Bisexual	Bachelor’s

The participants in this study came from a variety of backgrounds and experiences and held identities that included different sexual orientations. All participants identified as females in terms of sex and as women in terms of gender. Most of the participants were single with only one participant who was married. All participants attended 4-year medical programs within the midwestern U.S. and expected to receive an MD degree at the end of their medical schooling.

One participant was pursuing an MBA in addition to her MD degree. Many of the participants attended medical school in urban settings (72%), whereas the remaining participants attended medical school in a suburban setting (28%).

### Data analysis

Undergirded by BFT and LCFP, all authors engaged in some phase of the data analysis where we considered the importance of a sense of belonging. The data analysis occurred in four phases. First, the data was reviewed for errors after receipt from the transcription service. Then authors one and two performed an inductive analysis to identify initial themes relevant to the research question. We were particularly interested in whether the women felt like they belonged at their institution or ‘fit’ within the educational environment.

During the second phase of analysis, authors one and two performed a selective coding process where we asked several questions of the data to identify the most relevant themes to inform and shape the findings. For example, we asked questions such as: Who is a sense of belonging for? What does the institution (i.e., faculty and administration) do to indicate belonging? In what ways do students feel like they have power over their education? Can students be their authentic selves? In the third phase of analysis, we used BFT and LCFP to combine the selected codes and identify the final themes for this paper. The themes identified were not based on data saturation but were designed to highlight heterogeneity among the participants and identify whether the values held by the women were consistent with the values of their institution. Through the lens of BFT and LCFP, we understood that it was the institution’s role to create belonging and the participants’ place to sense that belonging. This understanding was key to how we analyzed the findings. In the final phase of analysis, author three, who joined the research team after the conclusion of the VC, interpreted the findings into composite narratives. The composite narratives creation process will be described in the following section.

### Creating the composite narratives

During the two-year period, the women in this study became more than just participants. Author one and two were able to build strong relationships with the women, relationships that remain today. Moreover, author three is a medical student who had regular interactions with a few of the participants. Therefore, for this study, we thought it appropriate to create composite narratives to highlight the experiences of the Latinas and Black women who participated in the research [37]. Through composite retellings, we maintain participants’ anonymity while using counterstorying to show interconnected yet distinct experiences of the Black women and Latina participants. We crafted separate counterstories for each group, capturing the nuances of being a Black woman or Latina. These counterstories unfold as a dialogue between a Black woman we named Moon and a Latina we named Star.

Each of the composites are based on the focus group and individual interview transcripts from the women in the study. Moon’s character is derived from three individual transcripts and the focus group materials from the Black women participants. Star is a composite of four Latinas’ interview transcripts and focus group materials. Some of the quotations and story elements such as character background came directly from the interview transcripts, while others were designed to move the story forward. Fictionalized setting details such as where the conversation took place are literary devices used to paint a picture and make the story feel complete. All real conversations for the study took place online.

To create the composite narratives, authors one and two provided author three with de-identified content from phase three of the data analysis. Author three was also provided guidance for how the story should be shaped as a conversation between two women. We decided to have this author produce the story due to their unbiased position as someone who did not know to whom the data belonged. Further, because of the unique position of author three as a medical student, we believed they could contextualize the findings in a story representative of how Black women and Latinas might behave. The story was completed after three drafts that were each reviewed and discussed amongst the research team to ensure accurate representation.

### Findings: the composite narrative

#### Moon

The door to the coffee shop jingled as Moon walked in and sat across from Star. It had been over a year since they last saw each other but medical school graduation was approaching, and the two friends wanted to catch up before they started their careers at residencies across the country.

“I can’t believe school’s almost over,” Moon said, waving the barista over to order a coffee. “I didn’t ever think I would make it here.”

Moon had struggled academically and socially in undergrad. With her grades, everyone including her friends, family, and advisors told her to give up on the dream of medical school.

With everyone around her constantly doubting her, she also began to doubt herself.

“These last few years have been a lot,” Star agreed.

From the start of medical school, I was just so happy to *get in* and be here, that it was easy to overlook things,” Moon replied. “But every time I look up or take a breath from studying, I feel so alone. Those times when I barely passed a test or failed a clinical assessment, it seemed like I was the only one struggling. I didn’t know who to talk to. No one looks like me at school, and no one seems to understand what I’m going through, where I’ve been, or where I was coming from.

You were pretty isolated in second year,” Star said. “I barely saw you outside of the library.

“I was always working so hard, trying to prove myself. I had to work twice as hard just to be seen. I spent all day trying to fit in, make the faculty like me, making everyone in my class like me. And then I would go home and try to tell my family about it but they didn’t understand. It was like being stuck in between two extremely different worlds. At school I had to have this bright cheery appearance, smile at patients, and make sure I spoke clearly and slowly.

Especially after an evaluation said that I was too “aggressive” when I spoke to patients.”

“Aggressive? That’s not like you at all,” said Star with a grimace.

I was one of the only Black women in my class and I always felt like I stood out. There were some nice faculty, the ones who wanted to get to know me better and help mentor me. But to some, I could tell they didn’t know what to do with me. It was never outright racist comments but microaggressions dropped in lectures as jokes at the expense of Black people. I just didn’t feel welcome here a lot of the time or at least like people were trying to include me.

“That’s rough,” Star said, nodding. “And what can you do? If you say something, you’re called defensive but if you don’t, people think you’re okay with it and they keep doing it. Plus, you’re at the bottom of the hierarchy as a medical student. You have more to lose if people think you’re difficult to work with.”

I think of this as being bigger than me,” Moon said. “My success makes it easier for the next Black person coming after me.

Moon was glad she was still here. She came into medical school with her best friend, another Black woman she’d grown up with but she and so many others from their class had dropped out along the way. Her friend’s mom got sick and when she was having trouble keeping up with the classwork, there weren’t enough resources to help keep her afloat. It was funny how her face had been on every medical school recruitment brochure and poster but when it was her turn to need help, no one was there. Each year, it seemed that someone from their class that Moon knew had to leave medical school and the farther she got along, the lonelier it got to look around and see less and less people who looked like her. She had to do what she could to make it to graduation. After all the sacrifices she and her community had made for her to be here, she had to see it through.

#### Star

Star blew on the top of her coffee before taking a sip, giving her time to take in what Moon had said. Star had felt just as much an outsider as Moon. She knew what it was like to struggle to find a support system. She was the daughter of Mexican immigrants who weren’t originally supportive of her dream to become a doctor. “I get where you’re coming from,” Star said. “I feel like medical school was harder for me than for some of my friends. Everything seemed to be always working against me. With my dyslexia, it was so hard for me to keep up with my small group and I couldn’t find anyone to study with because it took me longer to understand things. So, on top of being one of the only Latinas, I felt like no one wanted to work with me and it was embarrassing and lonely. I just stopped telling people about my dyslexia and tried to handle it on my own so people wouldn’t notice how different I was. I didn’t want people to treat me differently because I had a disability.”

Star had struggled academically for most of her life. Failing courses was normal for her in high school until college when she started getting the accommodations and study help that she needed.

Moon nodded. “I remember you telling me about how hard that was for you.”

“As if having a disability in medical school isn’t hard enough, right?” Star replied. “But then you have to add on worrying about all the racism and microaggressions while trying to be professional so you can manage how people view you. Did I tell you about this one preceptor I had? He was absolutely awful, but it wasn’t until one day when we were seeing a patient who was a man who worked in a factory with a hernia from lifting heavy boxes and the preceptor joked that the hernia meant he was actually working and that in his experience ‘the Hispanic patients don’t usually have jobs’.”

Moon inhaled sharply. “You’ve got to be kidding. A doctor said that? Out loud?”

I couldn’t believe it when I heard it. I looked around at the other students and no one said anything or even seemed disturbed by the comment which made me feel like I was overreacting. I wanted to raise my hand and say that one of those ‘Hispanics’ is standing right in front of you and would like an apology. But it was the same thing you were saying, I didn’t want to make him upset or make a scene. He was the one who would be evaluating me and a poor evaluation could impact my future residency options and career. In a way, I had to protect myself, even though I also feel the pressure to protect my culture. I tried to be diplomatic about my response, but it came out sounding sharper than I intended. I said something like the hardest working people I know are Hispanic and he just brushed it off as a joke.

“He really shouldn’t be treating patients if that’s how he treats people,” Moon said.

“I think it’s those instances of feeling excluded and like no one understood what I was going through that was why I had such a hard time finding where I belonged in medical school.

Some people don’t even know that I’m Latina until I tell them or until they see my name and then it’s like a light goes off in their brains and their face changes as they try to recategorize me.

And then you have the other hand where I walk into a patient room, and they automatically recognize me as Latina and get so excited. They’ll speak in Spanish so quickly and I have to do the awkward interjection that I only speak a little Spanish and watch their face fall with disappointment as I try to pull together sentences with the few words I do know. It’s like I’m too Latina to fit in with white people but not Latina enough at other times. My parents didn’t teach me Spanish, and we didn’t really do to much culturally growing up so I don’t really connect with that side of me in that way. Which makes it hard to connect with others in the community and also be the representative as a soon-to-be Latina physician I want to be.”

Star was still defining her identity as a Latina in medicine, a process that would continue after she graduated from medical school. She had expectations of the career she wanted, and those often diverged from what her family wanted for her, which was to pick a “woman’s” specialty and serve her community. They would have preferred she didn’t choose medicine at all, but now they urged her to focus on settling down, getting married, and building a family.

None of those were things that she wanted for herself, and she continued to work on finding her voice to advocate effectively for herself and others.

## Discussion

BFT and LCFP are key to our understanding concerning the importance of the themes highlighted in the composite narrative. These frameworks emphasize experiential narratives as high-quality data from which we can draw conclusions about key themes and challenges facing Black and Latina medical students. The themes identified through this composite fictionalized dialogue include the implications of intersectional identities and the need to establish rightful presence beyond simply belonging in a space. Given the nature of this work, these concepts (i.e., sense of belonging, intersectional identity, and rightful presence) connect to BFT and LCFP by offering analytical tools to understand how Black women and Latinas navigate predominantly white medical spaces, make sense of their intersectional identities, and describe how they have a right to occupy spaces without having to assimilate to dominate culture.

Intersectional Identity Development and Belonging Both Moon and Star’s stories illuminated the different ways Black women and Latina medical students in this study experienced medical education and how a sense of belonging impacted them. The stories exposed how Black women and Latinas are simultaneously navigating their personal and professional identity development while trying to determine if their identities belong in the medical school context. In Moon’s story, she mentioned how she was in search of individuals who looked like her, and how she actively worked on her impression management, or how others viewed her, by constructing a professional identity to fit in. Although Moon felt isolated in being the only one who looks like her, she also felt hyper visible because of the tropes ascribed to her identity as a Black woman. The experiences Moon recounted in the narrative are consistent with how literature describes Black women as both invisible and hyper visible in spaces where they are minoritized [23, 43]. Moon also shared how she was stuck between two worlds in how she had to show up in medical school versus how she could communicate with her family. Current literature supports Moon’s perception of how Black women negotiate their identity to shift to the spaces they occupy [44].

In Star’s narrative, she detailed the intersectionality of her multiple identities, explaining her Latin heritage and the intersection that comes with being a student with a disability. Star described how she was not fluent in Spanish or unfamiliar with certain Spanish customs, thus, revealing how she was still determining who she was as she navigated medical school. Star explained how her feelings of isolation based on comments made about individuals who shared her cultural background, and due to her disability, impacted her experience during medical school. She also detailed her journey of understanding her identity both in personal and professional settings.

Star and Moon’s stories have many similarities. Both reflected on feelings of being othered as a result of their minoritized status, and they both had trouble finding a community where they fit.

These findings are consistent with research that suggests URiM students must navigate professional identity formation in relation to the sociohistorical contexts of their personal identities [45]. The findings also confirm Madzia’s 2023 research that highlighted how URiM learners had to construct temporary identities that fit the school’s definition of professional [46].

Because the women must navigate complex identity formation based on their experiences in medical school, little room is left for them to focus on a sense of belonging. Thus, it may be necessary to move beyond belonging toward rightful presence.

### Toward rightful presence

According to Calabrese Barton and Tan, rightful presence is a concept where the qualities individuals offer to educational environments are accepted and embraced within the institutional makeup [47]. This acceptance therefore requires no adjustments by the learner to fit in. When using rightful presence, it is understood that everyone brings their own cultural capital to a learning environment and should already have the rights required to positively exist in that space without having to overcome delegitimizing barriers [47]. Unfortunately, medical students who have stories like those of Star and Moon must seek acceptance through finding a sense of belonging and often face significant problems if they do not find the supportive community that helps them navigate medical school.

Moon’s story emphasized the large responsibility that she felt being a Black woman in medicine.

Moon had the responsibility to be a positive representative of her community, to not feed into negative stereotypes, and to pave the way for others like her to pursue careers in medicine.

Especially when friendships were lost along the way. These responsibilities informed her decisions and how she handled certain situations as a medical student. The high expectations are a challenge for her, but they also gave a sense of purpose to her work. She recounted the emotions that come up for her when a member of her community acknowledged her position and the work that goes into her achievements.

For Star, the composite illustrated how she considered herself as representational for other Latinas in medicine. Star also expressed wanting to speak up for Hispanic patients when they require defending. Considering the definition of a sense of belonging includes a shared sense of purpose with the institution, the term seems antithetical to the experiences of Star and Moon who express a purpose of representing and defending their minoritized communities. This is especially true when behaviors that are perceived as negative toward a particular community are accepted as part of the status quo, which was illustrated by the physician’s behavior (a real incident) in the composite narrative. Therefore, rightful presence seems a more appropriate fit because it would signal to Star that calling attention to this negative behavior is her right in this environment, rather than being viewed as something that could negatively impact her belonging.

### Implications

Research confirms the importance of diversity and fostering a sense of belonging for URiM students [2, 18, 48]. However, in much of the medical education research, URiM students are not disaggregated for the various ethnic groups that exist. Although medical education scholars have explored the needs of women in medicine [49], few scholars have paid specific attention to the experiences of Black women and Latinas in medicine. To avoid essentializing these groups by confirming the effectiveness of a sense of belonging for all, it is important to honor the racial, ethnic, and gender variations found among each student demographic by disaggregating [50].

We did this by sharing the composite narratives of the experiences Black women and Latinas offer. This is because failing to understand the experiences of Black women and Latinas hinders the creation of effective interventions, impacting their retention in medical schools and exacerbating health disparities in society. Therefore, an implication of this research is to encourage the development of more research studies that highlight the unique experiences of medical students who are often lumped together in the URiM category by disaggregating the data.

Rightful presence is a concept that acknowledges the value students bring to the academic medicine environment without a focus on trying to establish a sense of belonging [46]. Rightful presence is relevant to the Black women and Latina students in this study because their values often misalign with the values of HWIs of academic medicine. Yet, Black women and Latinas offer capital to the medical education learning environment upon which they can build their success. This is especially true since the Black women and Latinas in this study identified situations that were inconsistent with their values and the responsibility they felt for their communities. Therefore, we encourage HWIs of academic medicine to move beyond a sense of belonging toward concepts of rightful presence where they acknowledge the rights of minoritized students, like Black women and Latinas, to exist in academic medicine settings without the additional burden of trying to fit in personally and professionally.

Using rightful presence, Black women and Latinas can be emboldened to speak up on behalf of their communities and drive their own academic success because they hold the intuition necessary for navigating HWIs. As represented through the composite narrative discussion between Star and Moon, Latina and Black women medical students face unique social challenges different from their medical school peers. These challenges include racism, microaggressions, feelings of isolation, challenges to their intersectional identities, and a lack of social support. Medical school administrators, faculty, and staff have a responsibility to support the Black women and Latinas they recruit by creating a community with antiracist principles, decreasing barriers to students seeking accommodations, and building the infrastructure that supports quality opportunities for these students. However, medical school administrators, faculty, and staff will continue to have trouble in developing the necessary interventions if they consistently view Black women and Latinas as students who must ‘fit’ into the school’s professional culture, rather than accepting their rightful presence within that culture. By meeting Black women and Latina medical students where they are and learning what they offer, medical schools can work to improve the diversity of academic resources as well as the diversity of social support.

### Limitations

Concerning limitations, it must be noted that although the VC was designed to serve as an environment to promote a sense of belonging for women of color, the VC was not viewed in this way by all the women. The women viewed the VC differently because they did not all attend the same institution. Moreover, it was clear that even the women who attended the same institution still had trouble seeing the VC as a strategy promoted by the institution because it was not a required intervention to promote a sense of belonging for all students. Concerning the participants, we did not ask for their immigration status in the demographic survey. Doing so may have gleaned more information that could be relevant to how we analyzed the findings; however, we did not account for this information. That said, Star did offer that her parents were Mexican immigrants. This was because one participant shared that information when others did not.

Additionally, the composite narrative format, while protecting anonymity, also can conceal the nuances described by members of the VC. The data was ultimately presented as the sum of the two groups, which might miss some of the cultural differences within the Black and Latina medical student groups. For example, though the Star narrative synthesizes their experiences as one, participants from different Latin American countries may experience different challenges to belonging and their social presence within their communities.

## Conclusions

In this study, we utilized BFT and closely related LCFP as frameworks for understanding how Black women and Latinas navigated academic medicine within HWI of academic medicine.

Using data collected from a VC of seven women who identified as Black or Latina from medical education institutions across the midwestern US, we developed composite narratives to highlight the experiences of the participants in relation to how a sense of belonging could impact those experiences. The composite narratives highlighted the similarities and differences between these two groups of women as they navigated the established academic and social structures of medical school.

Though medical educators have been working for years to increase diverse representation in medicine and offer social support to underrepresented medical students, there is still much to be done to enhance these students’ sense of true belonging within the medical community.

Therefore, there is a need for continued study of Black and Latina experiences within medical education, where medical educators should work to establish a sense of rightful presence in medicine for these learners instead.

## Supplementary Information

Supplementary Material 1.

Supplementary Material 2.

## Data Availability

The qualitative interview data from this study has been shared in the composite narrative format to protect the anonymity of participants. This study was exempt by the Indiana University Institutional Review Board, Protocol #11323. As a part of this review process, all participants provided their informed consent to participate in this research.

## Abbreviations

VC: Virtual community
BFT: Black feminist thought
LCFP: Latina/Chicana feminist perspective
URiM: Underrepresented in medicine
HWI: Historically white institution
CECE: Culturally engaging campus environments
