# Supplementary material for: Why do I need to belong? Black women and Latinas navigate medical education beyond belonging toward rightful presence
Source: BMC Med Educ. 2025 Aug 27;25:1205. doi: 10.1186/s12909-025-07781-9 (PMC12382051; doi:10.1186/s12909-025-07781-9)
Supplement: Supplementary file 1 — Supplementary Material 1. [file 12909_2025_7781_MOESM1_ESM.docx]

**Focus Group Survey Protocol**

Pre-Med  [why did you choose to attend medical school and why did you choose your particular institution]

1. Describe your educational pathway toward pursuing a medical degree. What or whom influenced your interest in medicine? What motivated you to attend medical school?
2. What messages did you receive about attending medical school?
3. What concerns did you have regarding medical school?
4. Describe your interests in becoming a physician.
5. Why did you choose to attend this particular institution?; What attracted you?

Medical School

1. How would you describe your medical school experiences thus far? How would you describe your clinical experiences? Interacting with preceptors? Interacting with peers? What clinical expectations do you set for yourself?
2. Describe your out of clerkship experiences. Are you involved in organizations?
3. How would you describe the dating climate for yourself? on campus for black women/latina in general?
4. What does engagement look like between you and other women of color on campus?
5. How do students and residents manage relationships with partners and spouses while in school?

General

1. How would you describe a successful medical school experience? residency experience?
   1. What do you see as the biggest challenges or barriers to success for black women? Latinas? For you?
   2. What do you see as the benefits of attending medical school? For you?

1. What strategies are you involved with or implementing to ensure your success?

1. What challenges do residents and students currently face?

1. How do residents and students cope with experiencing microaggressions?

1. What study strategies does each group use for various step exams?

1. What resources or wellness strategies have you used or that you have at your disposal to help you navigate your medical school experience? How do you take care of yourself mentally, physically, spiritually.

1. What advice would you give to other Black women and Latinas coming after you who wish to become a physician?

1. How do you think Latinas and Black women’s experiences differ from other medical student and resident populations?

1. What role do race, gender, sexuality, and other identities play in your medical school experiences? Have you experienced discrimination based on your identity? How did you handle it?

1. Do you engage in social media? Explain what applications you use. What role does social media play in your life as a black woman or Latina?

1. Do you watch medical dramas? What shows? Do they influence how you understand yourself as a black woman or Latina in medicine? What do think about the images of Black women and Latinas in these dramas?

1. What responsibilities or obligations do you have beyond medical school (e.g. to siblings, family, children, job)

1. What are your post-training aspirations? How has your experience prepared you to deal with life after training?
