# Supplementary material for: Why do I need to belong? Black women and Latinas navigate medical education beyond belonging toward rightful presence
Source: BMC Med Educ. 2025 Aug 27;25:1205. doi: 10.1186/s12909-025-07781-9 (PMC12382051; doi:10.1186/s12909-025-07781-9)
Supplement: Supplementary file 2 — Supplementary Material 2. [file 12909_2025_7781_MOESM2_ESM.docx]

**Demographic Survey**

*Please fill in the blanks or circle/check the most appropriate answer for the following questions. You may leave responses blank if you do not prefer to answer a question.*

**What is your name?**   ______________________________________________________________

(Your name will not be used in public files. All public research documents will include pseudonyms.  I ask for your name for follow up purposes.)

**What is your email address?** _____________________________________

**What is your age?** _____________

**Please indicate the racial group you most closely identify (circle all that apply):**

a. Black or African American

b. Native American or American Indian

c. Asian or Asian American

d. Pacific Islander

e. White

f.  Hispanic or Latino

g. Multiracial/Mixed-race

**Please list all ethnic background with which you identify (e.g., Caribbean, Guatemalan, Native Hawaiian, Samoan):** ______________________________________________

**Please indicate your sex (e.g., male, female)?**   _________________________

**Please indicate your gender (e.g., man, woman, transgender)?**   _________________________

**Please indicate your sexual orientation (e.g., heterosexual, gay, lesbian, bisexual)?**   _________________________

**What is your marital status (e.g., single, married, divorced)?** __________________________

**What is your highest educational attainment level?**

1. Professional Degree ( M.D., Ph.D.)
2. Master’s Degree
3. Bachelor’s Degree

**What undergraduate institution do you attend? _______________________________________________**

**What undergraduate medical school do (did) you attend? _______________________________________________**

**What graduate medical institution do you attend (if applicable)? _______________________________________________**

**Please select your institution type:**

1. 4-year public college/university
2. 4-year private college/university
3. Other:_________________________

**Please describe the geographical setting of your institution:**

1. Urban
2. Rural
3. Suburban

**When did you begin your degree? __________________________**

**When do you anticipate completing your training? _______________________**

**What type of degree are you pursuing (MD, MD/PhD/DO)? ______________________________**

**What is your current enrollment status?**

a. Full-time

b. Part-time
